# Supplementary material for: Spatial-temporal analysis of pulmonary tuberculosis in Hubei Province, China, 2011–2021
Source: PLoS One. 2023 Feb 7;18(2):e0281479. doi: 10.1371/journal.pone.0281479 (PMC9904469; doi:10.1371/journal.pone.0281479)
Supplement: S1 Table — (DOCX) [file pone.0281479.s001.docx]

Supplementary Files:

PTB notification rates database of Hubei, China(2011-2021)

| YEAR | POPULATION | PTBNR | BCNR |
| --- | --- | --- | --- |
| 2011-average | 57237695 | 81.6595 | 45.6657 |
| 2012-average | 57580054 | 82.2820 | 37.0944 |
| 2013-average | 57790005 | 77.8335 | 32.4364 |
| 2014-average | 57990000 | 74.2956 | 30.0069 |
| 2015-average | 58159999 | 72.0530 | 27.9023 |
| 2016-average | 58547500 | 70.9714 | 26.4999 |
| 2017-average | 58849995 | 66.1444 | 22.1614 |
| 2018-average | 59020004 | 60.9878 | 22.8939 |
| 2019-average | 59169975 | 58.9995 | 26.8008 |
| 2020-average | 59271272 | 51.4819 | 26.0733 |
| 2021-average | 57752547 | 53.6253 | 30.5995 |

The monthly registered number of PTB cases in Hubei, China (2011–2021)

| DATE | 2011 | 2012 | 2013 | 2014 | 2015 | 2016 | 2017 | 2018 | 2019 | 2020 | 2021 |
| --- | --- | --- | --- | --- | --- | --- | --- | --- | --- | --- | --- |
| Jan | 2778 | 2682 | 3153 | 3267 | 2954 | 2668 | 2730 | 2605 | 2812 | 2160 | 2367 |
| Feb | 3175 | 4154 | 2698 | 2954 | 2343 | 2610 | 3244 | 2270 | 2261 | 700 | 1873 |
| Mar | 4838 | 4713 | 4440 | 4165 | 4083 | 4030 | 3829 | 3483 | 3484 | 1498 | 2855 |
| Apr | 4038 | 4190 | 4061 | 3714 | 3760 | 3546 | 3349 | 3293 | 3489 | 3100 | 2861 |
| May | 4337 | 4276 | 4008 | 3667 | 3602 | 3569 | 3472 | 3259 | 3101 | 2794 | 2639 |
| Jun | 4291 | 4077 | 3609 | 3812 | 3835 | 3734 | 3620 | 3210 | 2973 | 3210 | 2757 |
| Jul | 3720 | 4003 | 3745 | 3686 | 3553 | 3246 | 3239 | 3192 | 3259 | 3289 | 2862 |
| Aug | 3890 | 3943 | 3548 | 3441 | 3555 | 3639 | 3440 | 3056 | 2953 | 2849 | 2430 |
| Sep | 3663 | 3587 | 3875 | 3858 | 3357 | 3632 | 3330 | 3030 | 2682 | 2946 | 2567 |
| Oct | 3915 | 3566 | 3781 | 3361 | 3495 | 3496 | 2687 | 2661 | 2760 | 2648 | 2345 |
| Nov | 4281 | 3996 | 4010 | 3516 | 3646 | 3833 | 3041 | 3015 | 2586 | 2730 | 2510 |
| Dec | 3814 | 4191 | 4052 | 3643 | 3723 | 3549 | 2945 | 2921 | 2550 | 2590 | 2904 |
