# Supplementary material for: Spatial-temporal analysis of pulmonary tuberculosis in Hubei Province, China, 2011–2021
Source: PLoS One. 2023 Feb 7;18(2):e0281479. doi: 10.1371/journal.pone.0281479 (PMC9904469; doi:10.1371/journal.pone.0281479)
Supplement: Attachment — Submitted filename: Response to Reviewers.docx [file pone.0281479.s002.docx]

List of Responses

Dear Dr. Li and Reviewers:

Thank you for your letter and for the reviewers’ comments concerning our manuscript entitled “Spatial-temporal analysis of pulmonary tuberculosis in Hubei Province, China, 2011-2021” (PONE-D-22-10280). Those comments are all valuable and very helpful for revising and improving our paper, as well as the important guiding significance to our researches. We have studied comments carefully and have made correction which we hope meet with approval.

Revised portion are marked in red in the paper. The responds to the reviewer’s comments are as flowing:

Reviewer #1:

1. Response to comment: In discussion part, the author thought the prevalence of TB in Hubei has been controlled to a certain extent. However, it could be biased if it was judged only by decline of notification rate. It may be due to low detection of PTB cases. More data was needed to this conclusion.

Response: It is really as Reviewer suggested that it could be biased to judge that the prevalence of TB in Hubei has been controlled to a certain extent only by the decline of notification rate. So I have removed the description, and described what the mainly effective measures were taken by government to control TB.

“It showed that the prevalence of PTB in Hubei province has been controlled to a certain extent.” was deleted.

2. For season trend, the author explained that most TB infections may occur in winter, with symptoms appearing after a certain time. However, for most adults in China, TB infection more likely occurred in previous years rather than recent one year. Thus this point was not reasonable. And also for “the impact of delayed diagnosis should also be considered”, what does it mean? Need much more explanation.

Response: As Reviewer suggested that most adults TB infection more likely occurred in previous years rather than recent one year. I know that the once infected, the individual is at highest risk of developing TB disease within the first two years, and among family contacts of TB patients, children younger than 5 years of age have a 19% chance of developing TB within 2 years. But I did not find relevant information about the probability of developing TB within one year after infection. So, to be more accurate, I've removed that sentence “thus most TB infections may occur in winter, with symptoms appearing after a certain time”.

About delayed diagnosis, I revised this part. In rural and remote areas of Hubei Province, most patients chose the nearest primary health facilities for their first medical consulation rather than visited TB designated hospitals directly, but the primary health facilities usually have no capacity or poor diagnosis of TB, and may misdiagnosed patients with common diseases such as cold or fever, leading to delayed in TB diagnosis, and consequently, more PTB cases were reported in summer seasons.

3. The study showed that Enshi Prefecture and Yichang city and Tongshan County in Xianning city were the areas with high notification rate of PTB in the past 11 years. Is there any change of hotspots for these regions during the 11 years? If there was some changes, it could be very useful to understand local TB control effects.

Response: Yes, Reviewer, there is any change of hotspots for these regions during the 11 years. I gave some descriptions in the manuscript. Little changed of hotspots in Enshi Prefecture, which has the highest TB epidemic in Hubei province. Changyang Tujia Autonomous County, Zigui County and Xingshan County, which were not all hot spots from 2011 to 2014. These counties are adjacent to Enshi Prefecture. This showed that there was weakness in TB control. The change of hot spots in Xianning was described in the manuscript, and the control measures are effective.

Line 321, “which were not all hot spots from 2011 to 2014.” was added.

Special thanks to you for your good comments.

Reviewer #2:

In the limitation section line 341 – 351 I feel the authors should include the fact that as far as I am aware they do not have access to any genotyping data or drug resistance data. I presume the majority of the patients received first line therapy and there may be potential for drug resistant clusters to spread as if undetected patients may fail the standard therapy, I would ask the authors to comment on this.

Response: It is really as Reviewer suggested that there may be potential for drug resistant clusters to spread. Unfortunately, in recent years, the proportion of drug-resistant tests in Enshi and Yichang has been increasing year by year, and the number of drug-resistant patients has also been increasing, but not all patients with active TB can be tested for drug sensitivity. Since we don't have comprehensive drug susceptibility test data, we didn’t do this analysis. This should be the foucus of our future efforts.

Line 362-364, “There may be potential for drug resistant clusters to spread. However, we did not perform this analysis because we did not have comprehensive drug susceptibility test data for all active TB patients.” was added.

Thank you very much for your good comments.

We tried our best to improve the manuscript and made some changes in the manuscript. These changes will not influence the content and framework of the paper. And we marked in red in revised paper.

We appreciate for Dr. Li and Reviewers’ warm work earnestly, and hope the correction will meet with approval. Thank you very much again for your suggestions and comments.

Yours sincerely.

Yu Zhang

Corresponding author:

Name: Liping Zhou

E-mail: zhouliping2268@163.com
